# First Report of the Gastropod-Killing Nematode, Phasmarhabditis californica, in Washington State, U.S.A

**DOI:** 10.2478/jofnem-2023-0013

**Published:** 2023-04-21

**Authors:** Rory J. Mc Donnell, Dana K. Howe, Dee R. Denver

**Affiliations:** Department of Crop and Soil Science, Oregon State University, Corvallis, OR 97331 Oregon USA; Department of Integrative Biology, Oregon State University, Corvallis, OR 97331 Oregon USA

**Keywords:** biological control, malacopathogenic nematode, *Phasmarhabditis californica*, snail, slug, survey, Washington

## Abstract

*Phasmarhabditis californica*, a commercially available biological control agent in England, Scotland, and Wales (Nemaslug 2.0 ®), was discovered for the first time in Washington State during 2022. Nematodes were recovered from the invasive gastropods *Arion hortensis, Deroceras reticulatum*, and *Oxychilus* sp. in garden centers in both Vancouver and Spokane. The 18S ribosomal RNA gene was used to identify the nematodes. This discovery builds upon previous reports of *P. californica* in California and Oregon and suggests that the species is widespread throughout the west coast of the U.S. Future research directions with *P. californica* are proposed.

*Phasmarhabditis* is the only nematode genus that has been commercialized as a biological control agent of pest slugs and snails (Morand et al., 2004). Of the 18 species currently known worldwide (Elena Ivanova and Jiřka Nermuť, pers. comms.), two are available as commercial products. The first, *Phasmarhabditis hermaphrodita*, formulated with a bacterial associate, *Moraxella osloensis* (Moraxellaceae), was launched as Nemaslug ® in 1994 and has been used broadly throughout Europe to control pest gastropods in a wide range of crops (Rae et al., 2007). Note that a recent study of Nemaslug ® by Sheehy et al. (2022) showed that the bacteria present were *Psychobacter* spp. and not *M. osloensis*_._ The second, *Phasmarhabditis californica* also formulated with *M. osloensis* (Stenberg et al., 2021), became commercially available in England, Scotland, and Wales as Nemaslug 2.0 ® in 2022.

Although past attempts at surveying for *Phasmarhabditis* in the United States were unsuccessful (Kaya, 2001; Ross et al., 2010), recent, more intensive searches resulted in the discovery of *P. hermaphrodita, P. californica, P. papillosa*, and an undescribed species closely related to *P. papillosa*, in California (Tandingan et al., 2014, 2016; Schurkmann et al., 2022). Similarly, in Oregon, *P. hermaphrodita, P. californica, P. papillosa*, and a second undescribed species have also recently been collected (Mc Donnell et al., 2018; Howe et al., 2020; Mc Donnell and Denver, unpubl. data). However, neither of the commercially available products are available for use in the U.S., as insufficient information exists on both the distribution of *P. hermaphrodita* and *P. californica* in other states and on their host range. In Canada, *P. californica* has been reported from Alberta (Brophy et al., 2020) but it is *P. hermaphrodita* that is available commercially, despite it never being collected in that country (NemaKnights Biological Slug Control).

Here, we report the first records of *P. californica* from Washington State on the west coast of the U.S. The nematodes were recovered from pest slugs and snails collected in garden centers in two locations (Table 1). Gastropod specimens were placed into 6 oz plastic containers with perforated lids with different containers used for each species and for each site. The host slugs and snails were identified using Mc Donnell et al. (2009) and Burke (2013). Containers were checked daily, and dead slugs and snails were removed and examined under a microscope for the presence of nematodes. Nematodes were transferred to an NGM agar plate and allowed to grow and reproduce on the plate, eating bacteria that co-cultured with the nematodes (Barrière and Félix, 2006). Molecular methods were used to identify the nematodes collected from the gastropod carcasses. PCR amplification and subsequent direct DNA sequencing of an ~800 bp segment of the nematode 18S ribosomal RNA gene revealed sequences (Accession # OQ584491 - OQ584496) that were a 100% match to 18S rRNA sequences for *P. californica* in GenBank (Accession # KM510210).

At the Vancouver site, we collected *Deroceras reticulatum* (3 individuals), *Ambigolimax valentianus* (3), *Arion hortensis* (11), *Deroceras laeve* (8), *Oxychilus* sp. (11) and *Succinea* sp. (3). *Phasmarhabditis californica* was found in one of the *D. reticulatum* and two of the *Oxychilus* specimens (overall recovery rate of 7.7%). In Spokane, we collected *D. reticulatum* (5), *A. hortensis* (5), *D. laeve* (4), *Oxychilus* sp. (8), and *Succinea* sp. (1). *Phasmarhabditis californica* was found in two of the *D. reticulatum* and one of the *A. hortensis* specimens (overall recovery rate of 13%).

Over the past 20 years, surveys for and research on *Phasmarhabditis* have intensified throughout the world, and this pattern is set to continue, given the interest in developing these nematodes as biological control agents. The results described here, building upon previous discoveries in California and Oregon, suggest that *P. californica* is widespread throughout the west coast of the U.S. It has also been collected in Canada (Brophy et al., 2020), New Zealand (Wilson et al., 2016), Ireland (Carnaghi et al., 2017), Wales (Andrus & Rae, 2019), and Germany (Keyte et al., 2022). We recommend surveying in other regions of the U.S. and other countries to determine the geographic range of *P. hermaphrodita, P. californica, P. papillosa*, and the recently discovered undescribed species. Given the concern among malacologists about using these nematodes as biocontrol agents and possible non-target effects (Christensen et al., 2021), we also recommend prioritizing research on the host range of the various species of *Phasmarhabditis*, particularly their potential impact on native malacofauna. Past surveys in the U.S. have focused exclusively on invasive gastropods, but we recommend including native slugs and snails in future efforts in order to determine if these malacopathogenic nematodes are infesting and currently impacting native, non-pest species.

**Table 1:** Date, location, gastropod host information, and GenBank Accession Numbers for *Phasmarhabditis californica* specimens collected in Washington State.

Date^a^	Host	Street	City, ZIP	County	Latitude	Longitude	GenBank No.
27 Apr 2022	*Deroceras reticulatum*	10006 SE Mill Plain Blvd	Vancouver, WA 98664	Clark	45.62160	-122.56996	OQ584492
27 Apr 2022	*Oxychilus sp*.	10006 SE Mill Plain Blvd	Vancouver, WA 98664	Clark	45.62160	-122.56996	OQ584491
27 Apr 2022	*Oxychilus sp*.	10006 SE Mill Plain Blvd	Vancouver, WA 98664	Clark	45.62160	-122.56996	OQ584494
30 Apr 2022	*Deroceras reticulatum*	2628 W Northwest Blvd	Spokane, WA 99205	Spokane	47.56769	-117.44557	OS584493
30 Apr 2022	*Deroceras reticulatum*	2628 W Northwest Blvd	Spokane, WA 99205	Spokane	47.56769	-117.44557	OQ584496
30 Apr 2022	*Arion hortensis*	2628 W Northwest Blvd	Spokane, WA 99205	Spokane	47.56769	-117.44557	OQ584495

^a^date gastropods were collected
